# The Impact of Magnetoencephalography-Directed Stereo-Electroencephalography Depth Electrode Implantation on Seizure Control Outcome in Children

**DOI:** 10.7759/cureus.29860

**Published:** 2022-10-03

**Authors:** Khashayar Mozaffari, Katherine Hofmann, Paul Boyd, Eric Chalif, Archana Pasupuleti, William D Gaillard, Chima Oluigbo

**Affiliations:** 1 Department of Neurosurgery, George Washington University School of Medicine and Health Sciences, Washington, DC, USA; 2 Department of Neurosurgery, Children's National Hospital, Washington, DC, USA; 3 Department of Neurology, Children’s National Hospital, Washington, DC, USA; 4 Department of Neurology, Children's National Hospital, Washington, DC, USA; 5 Department of Neurosurgery, Children’s National Hospital, Washington, DC, USA

**Keywords:** pediatric care outcomes, stereo-encephalography, magnetoencephalography, pediatric seizure, childhood epilepsy

## Abstract

Introduction

The use of magnetoencephalography (MEG) in localizing epileptic foci and directing surgical treatment of medically refractory epilepsy is well established in clinical practice; however, it has not yet been incorporated into the routine planning of stereo-electroencephalography (EEG) (SEEG) depth electrode trajectories during invasive intracranial evaluation for epileptic foci localization. In this study, we assess the impact of MEG-directed SEEG on seizure outcomes in a pediatric cohort.

Methods

A retrospective analysis was performed on a single-institution cohort of pediatric patients with medically refractory epilepsy who underwent epilepsy surgery. The primary endpoint was the reduction in seizure burden as determined by dichotomized Engel scores (favorable outcome: Engel scores I and II; poor outcome: Engel scores III and IV).

Results

Thirty-seven patients met the inclusion criteria (24 males and 13 females). The median age at seizure onset was three years, the median age at surgery was 14.1 years, and the median follow-up length was 30.8 months. Concordance was noted in 7/10 (70%) patients who received MEG-directed SEEG. Good clinical outcomes were achieved in 70% of MEG-directed SEEG patients, compared to 59.4% in their counterparts; however, this difference was not statistically significant (p=0.72). We noted no statistically significant association between sex, disease laterality, or age at surgery and good clinical outcomes.

Conclusions

Patients who underwent MEG-directed SEEG had favorable clinical outcomes, which demonstrated the practicability of this technique for determining SEEG electrode placement. Although no significant difference in clinical outcomes was obtained between the two groups, this may have been due to low statistical power. Future prospective, multi-institutional investigations to assess the benefit of MEG-directed SEEG are warranted.

## Introduction

Epilepsy is a disorder characterized by abnormal and excessive neuronal discharges that disrupt normal brain function [1]. Approximately 1.2% of the US population reports an active epilepsy diagnosis at any point in time, including a significant medical and social burden in the pediatric population [1,2]. Most patients with epilepsy can be managed with antiepileptic drugs (AEDs); however, approximately one-third of patients suffer from pharmacoresistant epilepsy [3,4]. In such cases, surgery remains the best treatment modality for decreasing seizure burden [5].

Prior to epilepsy surgery, patients undergo a series of imaging and electrographic studies to localize the epileptic foci [6]. Standard non-invasive methods include electroencephalography (EEG), magnetoencephalography (MEG), magnetic resonance imaging (MRI), fluorodeoxyglucose-positron emission tomography (FDG-PET), and single-photon emission computed tomography (SPECT) [6-8]. Multiple testing modalities are often necessary to accurately localize the epileptic foci [6-8] and eloquent areas [9,10]. In addition to the non-invasive modalities, invasive intracranial studies such as stereo-electroencephalography (SEEG) and other intracranial electroencephalography (iEEG), such as subdural electrodes (SDE), may be indicated if non-invasive investigations are unable to definitively confirm the seizure onset zone. These invasive intracranial investigations can provide further information [11], as they allow for direct measurement of electrical activity within the brain [11]. SDE leads are placed directly on the external cortical surface and measure a grid of electrical potentials, while SEEG or depth electrodes are linear implants that provide a superficial-to-deep montage of activity [11]. These iEEG methods collectively allow for a more precise measurement of neurologic activity, as the electrical potentials do not need to travel through bone or brain tissue to reach the external sensors utilized in non-invasive EEG [11].

First described in 1972 [12], non-invasive MEG utilizes a series of highly sensitive coils to measure the sum of magnetic fields generated by a subject’s brain. These measurements can then be mapped across a three-dimensional (3D) rendering of the cortex [13]. Many studies have shown the benefit of MEG in the context of epilepsy surgery [14-19]. Zhang and colleagues demonstrated that concordant findings between MEG and MRI in patients with localizable epileptic foci were linked with positive post-surgical results [20]. MEG has also been shown to have concordance with iEEG [14,15]. Additionally, MEG can detect foci smaller than the 18-20 cm^2^ needed for scalp EEG measurements [14,15] and does not require the precision necessary for iEEG placement [15]. MEG and SEEG concordance has been associated with favorable outcomes in epilepsy surgery patients [17-19,21,22]. Despite the growing use of MEG in the presurgical workup of patients with epilepsy [14], its efficacy is still debated, and there is a dearth of literature in the pediatric population. Herein, we aim to report our institution’s experience utilizing MEG to localize the epileptic focus and evaluate its utility in surgical planning for pediatric epilepsy patients.

## Materials and methods

Study design

A retrospective chart review and analysis were performed on patients with epilepsy referred for surgical management. Institutional review board approval was obtained (Pro00003724), and clinical data was obtained from the electronic medical records. A total waiver of consent for participants was obtained, and institutional review board approval from the Children’s National Hospital was granted (Pro00003724). Parameters of interest included age, sex, age at seizure onset, age at surgery, diagnostic studies, number of previous AEDs, number of prior surgeries for epilepsy, postoperative complications, and follow-up length. Patients with a follow-up length of less than one year were excluded. The Engel score classification [23] was utilized to assess surgical outcomes. Engel scores I and II were considered good outcomes, and Engel scores III and IV were deemed as poor outcomes. Prior to surgery, a multidisciplinary epilepsy team including neurologists and neurosurgeons was utilized to determine surgical appropriateness and the surgical approach. Phase 1 investigations, consisting of ictal video EEG (VEEG), 3T epilepsy protocol MRI, FDG-PET, functional MRI (fMRI), CURRY, and MEG, were performed, and whether to proceed to invasive intracranial monitoring using SEEG was considered by the interdisciplinary team when the phase 1 investigation results were deemed insufficient to localize the seizure foci definitively. The SEEG depth electrode implantation sites, determined by the multidisciplinary epilepsy team, were based on an anatomical-electrical-clinical hypothesis of the patient’s seizure onset and propagation. In addition, the region of the brain where a dipole was identified on MEG was selected as an additional site for the implantation of a depth electrode.

MEG data analysis

**Figure 1:**
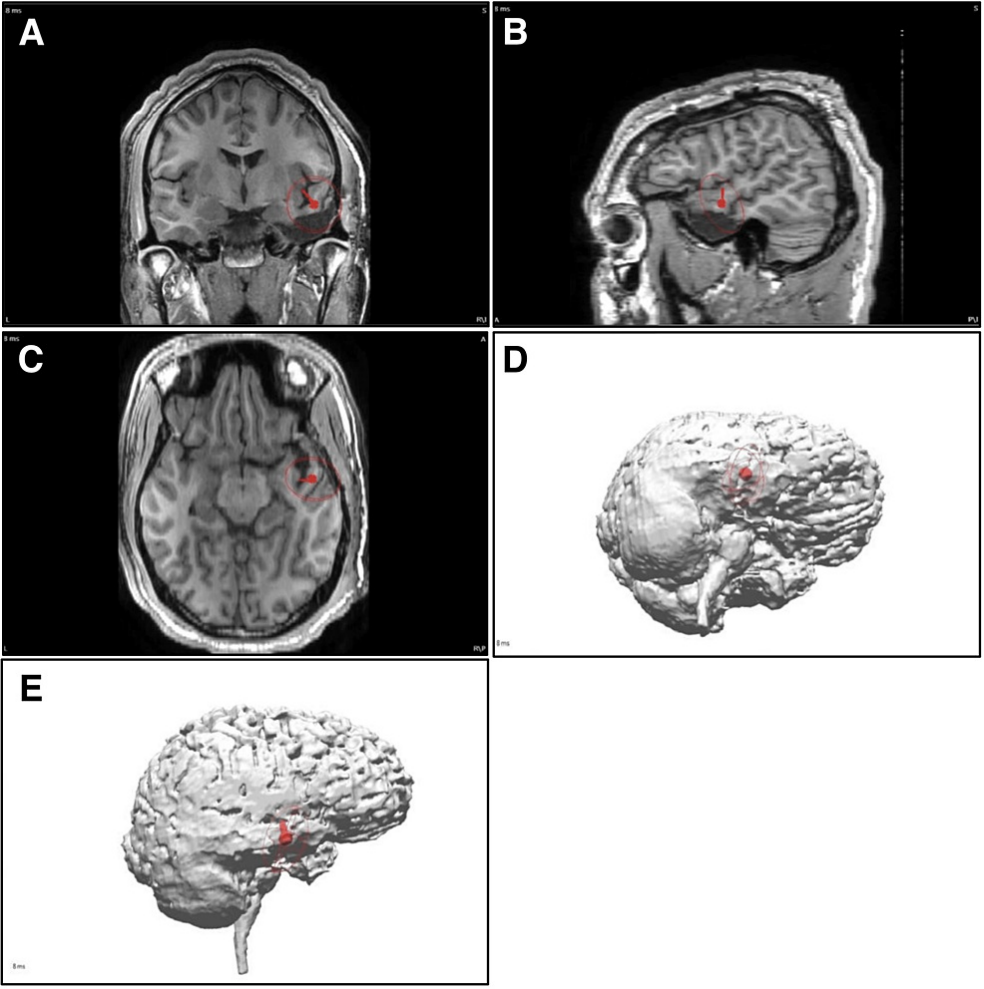
Illustration of the MEG findings for an 18-year-old male: MEG results identified right temporal dipole (A-C) Anatomical two-dimensional MRI images (right side is on the right). (D, E) Three-dimensional images depicting the dipole’s projection to the cortical surface MEG: magnetoencephalography; MRI: magnetic resonance imaging

MEG data was acquired using a 306-channel Elekta Neuromag® TRIUX system (Elekta AB, Stockholm, Sweden) in a magnetically shielded room with internal active shielding (IAS). A 25-channel electrode array was utilized to obtain simultaneous EEG recordings, using the international 10-20 system [24] with bilateral sub-temporal electrodes and CPz as a reference. The datasets were then filtered to exclude the external noise and further screened using appropriate low- and high-pass filters. Areas that contained visible epileptiform activity were identified for analysis of individual spikes. The Neuromag® TRIUX system software (Elekta AB, Stockholm, Sweden) was used to calculate the location, orientation, and strength of dipole sources. The patient’s MRI was referenced to the individually localized epileptic spikes to determine the anatomical location of the discharges. Although MEG is often presented as a collection of dipoles, it can also be reported as an average of the dipoles, especially if scattering is observed [25,26]. This study produced MEG dipole localization information as an average of the dipole clusters, superimposed on the patient’s co-registered preoperative two-dimensional (2D) and 3D MRI (Figure 1).

Determination of MEG and SEEG concordance

The anatomical location of the dipole clusters was exported via the Neuromag® TRIUX software. Subsequently, we gathered three radiographic studies: the preoperative MRI with overlayed dipoles, computed tomography (CT) obtained following SEEG implantation, and postoperative MRI. All SEEG contacts were manually illustrated on the CT image, which was superimposed on the postoperative MRI. The final clinical reports determined the regions of ictal onset and interictal activity on SEEG. If the SEEG contacts of interest were located within the same sulcus as the MEG cluster or within the gyri adjacent to the sulcus where the MEG cluster was located, such localization was considered concordant. If this overlap was not seen, the foci were deemed non-concordant. In cases of MEG and SEEG concordance, complete surgical resection of the foci identified by MEG was performed. In non-concordance cases, the foci identified by SEEG were resected.

Statistical analysis

Descriptive statistics were used to report patient outcomes. The mean and range are presented for select parametric continuous variables, and medians with corresponding interquartile ranges (IQR) are reported for nonparametric continuous variables. Categorical variables are presented as frequency (percentage). The predictability of the independent variables with several outcomes of interest was determined using univariate logistic regression. All statistical analysis was performed using RStudio v1.1.453 (RStudio, Boston, MA). A p-value of <0.05 was considered statistically significant.

## Results

Patient demographics

The summary of patient demographics is listed in Table 1. Selected demographics delineated by patient treatment group are listed in Table 2. Thirty-seven patients were included in our cohort and underwent epilepsy surgery. There were 24 males and 13 females. The median age at seizure onset was three years (IQR: 0.5-7.0). The median duration of seizures prior to surgery was 7.7 years (IQR: 4.2-11.6). Age at time of surgery had a median of 14.1 years (IQR: 7.2-16.6). The median follow-up length was 30.8 months (IQR: 17.8-42.2).

**Table 1:** Summary of patient demographics Median values are reported along with the interquartile range (IQR) AEDs: antiepileptic drugs; SEEG: stereo-electroencephalography

Variable	
Sex	24 males; 13 females
Age at time of seizure onset, years (IQR)	3.0 (0.5-7.0)
Age at time of surgery, years (IQR)	14.1 (7.2-16.6)
Duration of seizures prior to surgery, years (IQR)	7.7 (4.2-11.6)
Number of AEDs failed prior to surgery (IQR)	3.0 (2.0-5.0)
Length of SEEG implantation, days (IQR)	6.0 (4.0-8.5)
Follow-up length, months (IQR)	30.8 (17.8-42.2)

**Table 2:** Demographics by patient treatment group MEG: magnetoencephalography; SEEG: stereo-electroencephalography

Variable	MEG-directed SEEG (n=10)	All others (n=27)
Sex	Eight males; two females	16 males; 11 females
Mean age at time of surgery, years	12.7	11.8
Number of patients with good outcomes (Engel score I or II)	Seven patients (70%)	16 patients (59.4%)

The number of antiepileptic drugs utilized before surgery ranged from zero to 14, with a median of three drugs. The average number of presurgical studies performed was three (range: 0-7). A preoperative MRI and at least one VEEG were performed for every patient. Functional MRI (fMRI) and PET studies were performed in 56.8% and 48.6% of patients, respectively, and CURRY was utilized in the evaluation of two patients (5.4%).

The MRI and pathological findings of the cohort were analyzed. Cortical dysplasia was found in 27.0% of patients. In the patients, 13.5% were determined to have tuberous sclerosis. Injury due to encephalomalacia represented 10.8% of patients, while injury due to tumor represented 5.4% of patients, and injury due to volume loss was found in one patient (2.7%). In the cohort, 8.11% was found to have mesial temporal sclerosis, and 5.4% demonstrated hippocampal dysgenesis. Seven patients (18.9%) had unremarkable imaging and pathology, five of which were part of the MEG-directed cohort (Table 3).

**Table 3:** Imaging history and pathology/MRI findings by patient treatment group MRI: magnetic resonance imaging; VEEG: video electroencephalography; PET: positron emission tomography; MEG: magnetoencephalography; SEEG: stereo-electroencephalography; fMRI: functional MRI

	MEG-directed SEEG (n=10); number of patients (%)	All others (n=27); number of patients (%)
Imaging history		
VEEG	10 (100%)	27 (100%)
PET	7 (70%)	11 (40.7%)
Brain MRI	10 (100%)	27 (100%)
fMRI	7 (70%)	14 (51.8%)
CURRY	0	2 (7.4%)
Pathology/MRI findings		
Cortical dysplasia	3 (30%)	7 (25.9%)
Mesial temporal sclerosis	1 (10%)	2 (7.4%)
Tuberous sclerosis	1 (10%)	4 (14.8%)
Hippocampal dysgenesis	0	2 (7.4%)
Tumor injury	0	2 (7.4%)
Volume loss injury	0	1 (3.7%)
Encephalomalacia injury	0	4 (14.8%)
Heterotopia/polymicrogyria	1 (10%)	2 (7.4%)
Unremarkable	4 (40%)	3 (11.1%)

Fifteen patients underwent MEG (40.5%), and ten of those patients had MEG-directed SEEG. Among the MEG-directed SEEG cohort, the average age at the time of surgery was 12.7 years; patients who did not undergo MEG-directed SEEG had an average age of 11.8 years. In our cohort, a 70% (7/10) concordance rate was observed among the patients that underwent MEG-directed SEEG.

Clinical outcomes

In the entire cohort, good clinical outcomes were noted in 23 patients (62.2%), while poor outcomes were reported in 14 patients (37.8%). Although a higher percentage of positive clinical outcomes was observed in the MEG-directed SEEG group compared to their counterparts (70% versus 59.25%), this difference was not statistically significant (p=0.72). Within the MEG-directed SEEG cohort, 7/10 (70%) concordance between MEG and SEEG was noted. Among this subgroup, 5/7 (71.4%) of those with concordance noted favorable outcomes, while 2/3 (66.6%) of those with a lack of concordance achieved similar outcomes. This difference was not statistically significant (p=0.90).

Sex, age at the time of surgery, and the duration of seizures prior to surgery were not correlated with better outcomes. Table 4 demonstrates the results of logistic regression analyses that evaluated the association between these variables and seizure outcome.

**Table 4:** Statistical association between independent variables and good clinical outcome (Engel scores I and II) ^a^Evaluated whether “MEG-directed SEEG” was a predictor of good clinical outcomes compared to those whose SEEG electrodes were not directed by MEG. ^b^Evaluated whether among patients who underwent “MEG-directed SEEG (n=10),” concordance between MEG and SEEG was a predictor of good clinical outcomes MEG: magnetoencephalography; SEEG: stereo-electroencephalography

Variable	Odds ratio (95% CI)	P-value
MEG-directed SEEG^a^	1.58 (0.28-11.60)	0.72
MEG-SEEG concordance^b^	0.80 (0.04-23.5)	0.90
Male sex	0.62 (0.14-2.52)	0.50
Longer duration of seizures prior to surgery	0.96 (0.81-1.12)	0.60
Older age at surgery	1.06 (0.94-1.20)	0.30

## Discussion

Epilepsy is among the most common pediatric neurologic disorders, affecting approximately 1.2% of children [2]. Although most pediatric epilepsy patients can be managed with AEDs [2,3], those who fail pharmacotherapy require surgical intervention [5]. Various non-invasive imaging modalities have been utilized to accurately localize epileptic foci [9,27]. Among these, MEG has demonstrated applicability [9] and serves as a good supplement to preoperative planning before SEEG placement, which may be used to confirm the epileptic foci [14]. The present study evaluated the congruence between MEG and SEEG and the impact of such congruence on patients’ outcomes. In our cohort, we noted a great rate of concordance between MEG and SEEG, and patients who underwent MEG-directed SEEG had favorable clinical outcomes, demonstrating the practicability of this technique for determining SEEG electrode placement.

MEG and SEEG concordance has been shown to lead to a significant increase in seizure-free outcomes in multiple reports [17-19,21,22]. In a series of 47 patients (age range: 11-55 years), Liu et al. observed a 79% anatomical concordance between MEG and iEEG [19]. Within this cohort, the concordant group had a significantly higher percentage of seizure-free patients than the discordant group (51% versus 30%) [19]. Similarly, in a prospective study of 57 patients with non-lesional epilepsy (age range: 8-50 years), Mohamed et al. evaluated the usefulness of magnetic source imaging (MSI) (a non-invasive test that incorporates MEG and MRI results) [28]. Highly localized MSI results were associated with better surgical outcomes (83% versus 72% in the non-localized cohort), with a mean follow-up length of nearly six years [28]. In an exclusively pediatric population, Tenney et al. found that MEG had an accuracy of 75%-89.5% across various source identification algorithms compared to iEEG [22]. Additionally, they found that MEG-iEEG concordance was associated with better post-surgical outcomes [22]. Such studies demonstrate the improved outcomes with MEG and iEEG concordance and highlight the clinical application of this non-invasive modality [4,19,21,22,28].

In our series, pediatric patients underwent surgical resection of epileptic foci; MEG-directed SEEG was utilized in 10 patients, and there was concordance in 7/10 (70%) of these cases. Additionally, a higher percentage of patients in the MEG-directed SEEG cohort had good clinical outcomes compared to those who did not undergo MEG-directed SEEG (70% versus 59.25%). However, univariate analysis did not reveal a significant association between MEG-SEEG concordance and more favorable Engel scores. Nonetheless, these results demonstrate a greater seizure reduction rate within the MEG-directed SEEG group. Despite the lack of statistical significance, which was likely due to the limited statistical power of the study, this report, along with the aforementioned studies [12,22,24], underscores the utility of MEG in guiding SEEG and subsequently increasing the likelihood of achieving favorable outcomes in epilepsy patients. Additionally, our study was unique in that we evaluated an exclusively pediatric cohort of patients. Such findings are an important addition to the existing literature as there is a scarcity of literature in this patient population concerning MEG and SEEG.

Limitations

The strengths and limitations of this study should be acknowledged. All operations were performed by the same neurosurgeon with consistent operative techniques, thus reducing performance bias. This study was also a relatively large cohort of patients receiving both SEEG and MEG with long-term (≥1 year) results. Due to the retrospective nature of this study, it is limited by a lack of control groups or blinding. As a single-institution evaluation, our cohort contains only 15 patients who underwent MEG and only 10 MEG-directed SEEG patients. This significantly limited the statistical power of our study. Additionally, the decision to perform MEG as a preoperative diagnostic study was based on the decision of our institution’s epilepsy multidisciplinary team, as our team performed MEG scans only in situations where seizure foci were still unclear after a comprehensive analysis of the seizure semiology and other phase 1 studies. This likely posed a selection bias, thus influencing the composition of patients in our series. Furthermore, this practice of selective MEG investigations in our center selected for patients in whom seizure focus localization was particularly challenging likely contributed to the low statistical power of the study. Additional prospective, blinded, multi-institutional studies are warranted to validate our findings and further investigate the utility of MEG in identifying the epileptogenic foci in pediatric patients.

## Conclusions

Overall, patients who received MEG-directed SEEG demonstrated a higher percentage of positive surgical outcomes compared to those not assessed in this manner, albeit the lack of statistical significance. Barring further study, MEG-directed SEEG should continue to be utilized as a part of a wider range of tests prior to epilepsy surgery. The use of MEG in determining SEEG electrode placement and targets could lead to improved clinical outcomes.
